# Elemental composition and bioaccessibility of farmed oysters (*Crassostrea gigas*) fed different ratios of dietary seaweed and microalgae during broodstock conditioning

**DOI:** 10.1002/fsn3.1044

**Published:** 2019-06-28

**Authors:** Carlos Cardoso, Romina Gomes, Ana Rato, Sandra Joaquim, Jorge Machado, José Fernando Gonçalves, Paulo Vaz‐Pires, Leonardo Magnoni, Domitília Matias, Inês Coelho, Inês Delgado, Isabel Castanheira, Joana Matos, Rodrigo Ozório, Narcisa Bandarra, Cláudia Afonso

**Affiliations:** ^1^ Division of Aquaculture and Upgrading Portuguese Institute for the Sea and Atmosphere, IPMA Lisboa Portugal; ^2^ Interdisciplinary Centre of Marine and Environmental Research (CIIMAR/CIMAR) University of Porto Matosinhos Portugal; ^3^ Department of Aquatic Production, Abel Salazar Biomedical Sciences Institute (ICBAS) University of Porto Rua Jorge de Viterbo Ferreira 228 4050‐313 Porto Portugal; ^4^ Food and Nutrition Department National Health Institute Doutor Ricardo Jorge (INSA, IP) Lisbon Portugal; ^5^ Faculdade de Ciências da Universidade de Lisboa Lisbon Portugal

**Keywords:** bioaccessibility, dietary effects, elemental composition, microalgae, Pacific oyster, seaweed

## Abstract

The Pacific oyster (*Crassostrea gigas*) culture has been expanding, thereby leading to a greater importance of hatcheries. Broodstock conditioning is very important in the hatchery process, in which diet composition may have a strong influence on the offspring production and quality. Therefore, the current study evaluated elemental composition and bioaccessibility of oysters fed different ratios of dietary seaweed (SW) and microalgae. The dietary conditioning consisted of direct replacement of microalgae by SW at four substitution levels (0%, 25%, 50%, and 100% diet). It was observed that oysters fed 100% SW had the highest levels of Be, Cu, Zn, Sr, and Cd. The most important trend was a concentration decline of most elements with progressively lower levels of SW substitution for microalgae in the feeds. No Cd or Pb hazard (contents below 1.0 mg/kg for Cd and 1.5 mg/kg for Pb) was found in oyster meat. Regarding elemental bioaccessibility, values were similar, near 100% in the cases of Cu, Br, and I. Only for Mn and Pb, bioaccessibility percentages deviated more from 100%. Indeed, the value for Pb was 50% ± 7% (initial group), and for Mn, all values were equal or lower than 29% ± 2% (final group of oysters fed microalgae). It was observed that Mn, Cd, and Pb bioaccessibility increased with a growing share of microalgal biomass in the feed. Therefore, this study showed that SW incorporation into the feed influences elemental composition and bioaccessibility of the oysters.

## 1 INTRODUCTION

Oyster (*Crassostrea gigas*) is a highly valorized seafood product. It is a marine bivalve mollusk belonging to the family Ostreidae. They are one of the best known and most widely cultivated marine animals. Its cultivation has greatly expanded worldwide, exceeding a production of 600,000 tonnes in 2014 (FAO, 2014). The production of oyster is sometimes dependent on spat collection from the wild (Chávez‐Villalba et al., 2002). However, it has been observed a great interannual variability in the wild, which, combined with an ever‐increasing demand for juveniles, has fostered the development of hatcheries.

Hatchery production encompasses three distinct stages: broodstock conditioning, larval culturing, and postlarval rearing phase. Broodstock conditioning is a stage of the utmost importance in hatchery, because it is the first chance to modulate/condition the whole offspring. A higher oyster fecundity, better quality eggs, and enhanced larval viability are possible through intelligent innovations in broodstock conditioning (González‐Araya, Lebrun, Quéré, & Robert, 2012; Utting & Millican, 1997). It has been shown that physical and nutritional factors can modulate gonadal development, either accelerating gametogenesis or slowing gonadal maturation (Gallager & Mann, 1986).

There are several nutritional studies on bivalve species that have tested different algal compositions for an enhanced reproductive outcome (Anjos et al., 2017; González‐Araya et al., 2012; González‐Araya, Quéau, Quéré, Moal, & Robert, 2011; Pronker, Nevejan, Peene, Geijsen, & Sorgeloos, 2008; Utting & Millican, 1997). Currently, the most successful approach to a balanced diet lies in microalgal blends. Due to its nutritional value, it is possible to obtain optimal food conditions (Brown & Robert, 2002). This entails a dependence on the production of live microalgae, which may represent up to 30%–40% of the hatchery operational costs (Coutteau & Sorgeloos, 1992). This economic limitation has fostered research focusing on dietary replacement of live microalgae in bivalve hatcheries (Arney, Liu, Forster, McKinley, & Pearce, 2015; Boeing, 1997). A research line has focused on seaweed (SW), which is deemed to be a food source both for human and animal nutrition, mainly due to its high nutritional value (Peng et al., 2015).

On the one hand, it is known that the dietary nutritional profile influences the physiology of bivalves, having typically a strong effect on proteins, carbohydrates, and lipids (Joaquim et al., 2011; Matias, Joaquim, Leitão, & Massapina, 2009). On the other hand, bivalves are known to be accumulators of mineral elements, including some contaminants. As a result, in the wild, oysters are an excellent source of iron (Fe), copper (Cu), zinc (Zn), and selenium (Se) (Noël et al., 2012). In oyster farming, elemental composition will reflect pond/tank water composition (besides feed formulation), that is, environmental influence is crucial (Bilandžić et al., 2014). Therefore, though oysters can contribute to achieving the recommended daily intake of some essential elements, they may also contribute to human exposure to environmental pollutants (Guérin et al., 2011). Moreover, oysters may serve as bioindicators for monitoring a variety of contaminants in the environment. It has been shown that oysters originating from contaminated sites usually display higher metal contents (Amiard et al., 2008).

Besides a relatively high protein content (10%–25% dry weight), some green seaweeds, such as the species of genus *Ulva*, also have high levels of mineral elements with nutritional value, including calcium and magnesium (Fleurence et al., 2012). Moreover, the utilization of *Ulva* species as a dietary ingredient has been shown to improve stress response and resistance to disease, thereby representing a meaningful advantage to aquaculture (Fleurence et al., 2012).

In order to evaluate the potential nutritional value of oyster, it is crucial to know whether any given nutrient becomes bioaccessible—that is, available for human intestinal absorption (Cardoso, Afonso, Lourenço, Costa, & Nunes, 2015)—after digestion. In this regard, it should be noted that oral bioavailability is defined as the fraction of an ingested contaminant in a certain matrix that reaches systemic circulation (Brandon et al., 2006), being three steps distinguishable: (a) release of the substance from its matrix during digestion in the gastrointestinal tract, GI (bioaccessibility), (b) absorption of the bioaccessible fraction, and (c) metabolism in the intestine and liver. Hence, bioaccessibility indicates the maximum fraction that is released from the food matrix into the digestive tract (mouth, stomach, and intestine) (Brandon et al., 2006). Over the last years, the use of in vitro methodologies to study a compound bioaccessibility in the human gastrointestinal tract has been considered a suitable tool and, within the proposed methods, the procedure described by Afonso et al. (2015) is considered practical and reliable.

In order to assess the effect of substituting SW on broodstock conditioning of *C. gigas*, the current study replaced a dietary control of live microalgae by commercial dry seaweed *U. rigida* at 0%, 25%, 50%, and 100% substitution. The elemental composition and bioaccessibility—as assessed by an in vitro digestion model—were studied, aiming to fully understand the physiological and nutritional implications of different diets in the broodstock conditioning stage.

## 2 MATERIAL AND METHODS

### 2.1 Experimental design

#### 2.1.1 Algal culture and diet formulation

Microalgae (Alg) *Tisochrysis lutea* (formerly known as *Isochrysis affinis galbana* or T.ISO), *Skeletonema costatum* (SKT), and *Chaetoceros calcitrans* (C‐Cal) were batch‐cultured in a plastic bag (80 L) with filtered (0.35 µm) and UV‐treated natural seawater. Seawater was chlorinated for 24 hr, neutralized with thiosulphate, and enriched with f/2 medium before inoculation. Microalgae were grown at a temperature of 18 ± 2°C, under continuous aeration, to improve growth and avoid algae settlement, and under constant conditions of light, at an intensity of 9,900 lux at the culture surface. Microalgae were harvested daily in the late‐exponential growth phase. Before being used as food, algal densities were determined, in a daily‐based, by resorting to standard algal cell counts (Bürker chamber). Microalgae blend was constituted of one third of T.ISO (size: 3 × 5 µm; dry weight: 30.5 pg) and two thirds of diatoms: 75% of SKT (size: 10 × 5 µm; dry weight: 52.2 pg) and 25% of C‐Cal (size: 3–6 µm; dry weight: 11.3 pg) (Brown, Jeffrey, Volkman, & Dunstan, 1997).

The seaweed (SW) *Ulva rigida* was cultivated for 4 months in a commercial SW production (AlgaPlus Lda.) using an integrated multi‐trophic aquaculture (IMTA) system with filtrated water from seabass and gilthead seabream aquaculture production. Seaweed was then oven‐dried and triturated.

Four nutritional regimes were tested: one nutritional regime was constituted by 100% microalgae (Alg) blend (Diet SW0), whereas the other three regimes were formulated with different proportions of commercial dry SW *Ulva rigida* (<150 µm): 25% SW and 75% Alg (Diet SW25); 50% SW and 50% Alg (Diet SW50); and 100% SW (Diet SW100).

#### 2.1.2 Feeding trial—Broodstock conditioning

Seven hundred and twenty adult oysters (83.45 ± 12.42 g total mean weight; 2.40 ± 0.77 g average dry meat weight; 10.4 ± 0.96 cm mean length) from Ria de Aveiro (Portugal western coast—40º42′N; 08º40′W) were equally and randomly distributed into four groups (*n* = 3 tanks/group), and each group was conditioned with one of the four nutritional regimes. A group of eight oysters were sampled before the start of the trial, constituting the group “Initial.” These oysters were carefully excised, and the entire soft tissue was removed, freeze‐dried, and stored at −80°C until analysis.

Experimental tanks contained natural seawater filtered in a flow‐through system at a flow rate of 0.8 L/min. Water salinity was 33 g/L, and water temperature was maintained at 21 ± 1°C by using heat exchangers. According to seaweed percentage in each diet, seaweed was weighed, resuspended in natural filtered seawater, and afterward added to the food supply tanks, with strong and continuous aerations to avoid deposition. Food was added daily to the circuit through a pump, at a ratio of 4% of oyster dry weight (g) in algal dry weight (mg) (Delaporte et al., 2006; Utting & Millican, 1997). In order to keep food ration constant, the amount of food was daily adjusted accordingly to total biomass in each experimental condition. The *C. gigas* broodstock were conditioned during a period of eleven weeks, from February 2017 to April 2017.

At the end of the trial, a group of 8 oysters from each nutritional regime were randomly selected and carefully excised, and the entire soft tissue was removed, freeze‐dried, and stored at −80°C until analysis.

### 2.2 In vitro digestion model

An in vitro digestion model was chosen for the determination of elemental bioaccessibility in each of the five oyster groups (Initial, SW100, SW50, SW25, and SW0). Such model comprises three sections, which enable the simulation of the human digestion in three different parts of the GI tract: mouth, stomach, and small intestine (Afonso et al., 2015).

The composition of digestive juices (saliva, gastric, duodenal, and bile) was the same as described by Afonso et al. (2015). The chemicals KCl, NaH_2_PO_4_, Na_2_SO_4_, NaCl, NaHCO_3_, HCl, CaCl_2_.2H_2_O, KH_2_PO_4,_ and MgCl_2_ used for preparation of the digestive fluids were obtained from Merck. NH_4_Cl was obtained from Fluka, and all other chemicals were obtained from Sigma (St. Louis). In the case of duodenal juice, trypsin and α‐chymotrypsin from Sigma (St. Louis) were also added.

Briefly, approximately 0.5 g homogenized freeze‐dried oyster meat was weighed. Sample was mixed with 4 ml of artificial saliva at a pH 6.8 ± 0.2 for 5 min, then 8 ml of artificial gastric juice (pH 1.3 ± 0.02 at 37 ± 2ºC) was added, and pH was lowered to 2.0 ± 0.1. The mixing lasted 2 hr in a head‐over‐heels movement (37 rpm at 37 ± 2ºC). Finally, 8 ml of artificial duodenal juice (pH 8.1 ± 0.2 at 37 ± 2ºC), 4 ml of bile (pH 8.2 ± 0.2 at 37 ± 2ºC), and 1.33 ml of HCO_3_^‐^ solution (1 M) were added. The pH of the mixture was set at 6.5 ± 0.5, and agitation for 2 hr was identical to gastric conditions. The mixture generated in the in vitro model was subjected to centrifugation at 2,750 *g* for 5 min, thus yielding a nondigested portion and the bioaccessible fraction. While chemicals were supplied by Merck, enzymes were attained from Sigma (St. Louis).

#### 2.2.1 Calculation of bioaccessibility

The percentage (%) of each oyster element (E) in the bioaccessible fraction only or in both the bioaccessible and in the nondigested fractions was estimated as follows:

Calculation of bioaccessible Mn, Cu, Sr, Cd, and Pb

$$\%\text{E bioaccessible}\;=\;{\left[E\right]}_{\text{bioaccessible}}\times 100/{\left[E\right]}_{\text{initial}}$$

Being:

$${\left[E\right]}_{\text{bioaccessible}}=\text{Concentration of element in the bioaccessible fraction of the sample;}{\left[E\right]}_{\text{initial}}\;\text{= Concentration of element before digestion}.$$

Calculation of bioaccessible and nondigested Br and I

$$\%\text{E bioaccessible =}\;{\left[E\right]}_{\text{bioaccessible}}\times 100/\left[S\right]$$

and

$$\%\text{E nondigested =}\;{\left[E\right]}_{\text{nondigested}}\times 100/\left[S\right]$$

Being:

$${\left[E\right]}_{\text{bioaccessible}}\;\text{= Concentration of element in the bioaccessible fraction of the sample;}{\left[E\right]}_{\text{nondigested}}\;\text{= Concentration of element in the nondigested fraction of the sample;}$$

$$\left[S\right]={\left[E\right]}_{\text{bioaccessible}}+{\left[E\right]}_{\text{nondigested}}$$

### 2.3 Analyses

#### 2.3.1 Mineral elements and contaminants

With the exception of Br and I, for elemental determination, freeze‐dried samples were weighed (0.5 g) in triplicate and digested using a closed‐vessel microwave digestion system (Milestone ETHOS 1 Series). Microwave digestion method was as specified in Nascimento et al. (2014). Samples were diluted to 25 ml with deionized water.

For Br and I determination, 0.2 g of freeze‐dried sample was weighed into a 50 ml tube. Extraction was obtained using a graphite block system for 3 hr at 90°C. All samples were centrifuged and filtered through 0.45 µm filters.

Elemental composition of the bioaccessible fraction was obtained by making a minimum 10‐fold dilution of this fraction using deionized water. Dilutions were adjusted according to elemental content.

Blank solutions and certified reference materials were prepared with the same procedure of the samples. Standard curves were used for the determination of analyzed elements. All elements were analyzed by an inductively coupled plasma mass spectrometer, ICP‐MS Thermo X series II (Thermo Fisher Scientific). ICP‐MS instrumental setting was as specified in Nascimento et al. (2014). Iodine was analyzed separately from the remaining elements. The elemental concentration was expressed in mg/kg dry weight.

#### 2.3.2 Quality control

A minimum of two replicate analyses were performed for each sample and analysis. All standards and reagents were of high purity (over 99.5%). To quantify elements by the ICP‐MS methods, a calibration curve was made with no less than five standards in different concentrations. Accuracy was checked by analysis of several standard reference materials (Table 1). From these results, it could be concluded that the analyzed material was in the value range of the certified material. The limits of quantification (LOQ) of the elements were the following ones: Li—0.01; Be—0.01; Cr—0.02; Mn—0.02; Co—0.01; Ni—0.02; Cu—0.02; Zn—0.25; As—0.01; Se—0.02; Br—0.61; Sr—0.02; Mo—0.02; Cd—0.01; Sn—0.01; I—0.06; Tl—0.01; and Pb—0.03 mg/kg.

**Table 1:** Laboratory performance on certified reference material (ERM‐CD200) (*n* ≥ 4)

Element (mg/kg)	Certified value	Present work
Cu	1.71 ± 0.18	1.88 ± 0.04
Zn	25.3 ± 1.7	25.0 ± 0.6
As	55 ± 4	47 ± 1
Cd	0.95 ± 0.06	0.92 ± 0.03
Pb	0.51 ± 0.06	0.58 ± 0.02

Note

Values are presented as average ± *SD*. ERM‐CD200—European Reference Material, Seaweed (*Fucus vesiculosus*) (Institute for Reference Materials and Measurements of the European Commission's Joint Research Centre).

### 2.4 Statistical analysis

Analyses were done in triplicate. The four nutritional regimes (SW100, SW50, SW25, and SW0) were compared as well as the five studied oyster groups (Initial, SW100, SW50, SW25, and SW0) before and after digestion. In order to test normality and variance homogeneity, Kolmogorov–Smirnov's test and Levene's *F* test, respectively, were used. Data fulfilled both of these parametric tests’ assumptions with exception of Co, Mo, and I in the oyster feeds and Cr, Mn, Co, Ni, Br, Mo, I, Tl, and Pb in the oysters. The parametric test, Tukey's HSD, was done with STATISTICA 6, 2003 version (StatSoft, Inc.). For all statistical tests, significance level (α) was 0.05. Regarding the nonparametric test, differences were analyzed with nonparametric analysis of variance (Kruskal–Wallis) followed by nonparametric multiple comparisons test (Zar, 1999).

## 3 RESULTS AND DISCUSSION

### 3.1 Feed elemental composition

The elemental composition of the four feeds used as diets during the broodstock conditioning of the oysters (SW100, SW50, SW25, and SW0) is displayed in Table 2.

**Table 2:** Elemental composition (mg/kg dry weight and µg/kg dry weight) of the used oyster feeds

Element	SW100	SW50	SW25	SW0
Li (mg/kg dw)	2.14 ± 0.03^a^	1.58 ± 0.01^b^	1.30 ± 0.01^c^	1.02 ± 0.01^d^
Be (µg/kg dw)	26 ± 1^a^	15 ± 1^b^	10 ± 2^c^	4 ± 2^d^
Cr (mg/kg dw)	3.36 ± 0.24^a^	4.05 ± 0.12^b^	4.39 ± 0.14^c^	4.73 ± 0.21^c^
Mn (mg/kg dw)	99.8 ± 3.5^a^	135.2 ± 2.0^b^	152.9 ± 2.0^c^	170.6 ± 2.6^d^
Co (µg/kg dw)	818 ± 15^a^	1,207 ± 8^ab^	1,401 ± 5^ab^	1,596 ± 3^b^
Ni (mg/kg dw)	4.73 ± 0.18^a^	2.85 ± 0.12^b^	1.90 ± 0.09^c^	0.96 ± 0.06^d^
Cu (mg/kg dw)	21.9 ± 0.1^a^	14.3 ± 0.1^b^	10.6 ± 0.1^c^	6.8 ± 0.1^d^
Zn (mg/kg dw)	8.02 ± 0.33^a^	22.81 ± 0.87^b^	30.21 ± 1.15^c^	37.60 ± 1.42^d^
As (mg/kg dw)	3.34 ± 0.18^a^	3.11 ± 0.21^a^	2.99 ± 0.23^a^	2.87 ± 0.25^a^
Se (mg/kg dw)	1.07 ± 0.11^a^	2.01 ± 0.03^b^	2.47 ± 0.08^c^	2.94 ± 0.14^d^
Br (mg/kg dw)	466 ± 5^a^	469 ± 9^a^	471 ± 11^a^	472 ± 15^a^
Sr (mg/kg dw)	53.8 ± 0.8^a^	117.1 ± 4.6^b^	148.7 ± 6.6^c^	180.3 ± 8.7^d^
Mo (mg/kg dw)	2.04 ± 0.10^a^	1.53 ± 0.04^a^	1.28 ± 0.01^a^	1.03 ± 0.02^a^
Cd (µg/kg dw)	26 ± 0^a^	37 ± 2^b^	42 ± 3^c^	48 ± 3^c^
Sn (µg/kg dw)	366 ± 9^a^	342 ± 6^ab^	331 ± 14^ab^	319 ± 21^b^
I (mg/kg dw)	44.6 ± 0.8^a^	23.6 ± 0.4^ab^	13.2 ± 0.1^ab^	2.7 ± 0.1^b^
Tl (µg/kg dw)	7 ± 0^a^	9 ± 1^ab^	10 ± 1^b^	11 ± 1^b^
Pb (mg/kg dw)	1.69 ± 0.11^a^	1.11 ± 0.06^b^	0.82 ± 0.04^c^	0.52 ± 0.02^d^

Note

Values are presented as average ± *SD*. Different letters within a row correspond to statistical differences (*p* < 0.05).

The studied elements, Li, Be, Cr, Mn, Co, Ni, Cu, Zn, As, Se, Br, Sr, Mo, Cd, Sn, I, Tl, and Pb (arranged in order of increasing atomic weights), enabled a thorough analysis of the elemental composition of the feeds. There were significant differences between the four feeds. The concentration variation between these different feeds was diverse: As, Br, and Mo content remained unchanged across the four feeds; for Cr, Mn, Co, Zn, Se, Sr, Cd, and Tl, higher levels of microalgal biomass in the feed led to higher elemental content; and for Li, Be, Ni, Cu, Sn, I, and Pb, lower levels of SW substitution for microalgae entailed lower elemental content. The variations were consistent, that is, contents increased or decreased progressively as a function of SW substitution degree in the feed.

The attained results were largely affected by the fact that both microalgae (T.ISO, SKT, and C‐CAL) and SW (*U. rigida*) were cultivated, thus being their elemental composition determined by the cultivation conditions. A typical feature of the elemental composition of SW, high I content, was also observed. Indeed, the I content of the SW100 feed, 44.6 ± 0.8 mg/kg dw, did not differ much from those of the literature (Nitschke & Stengel, 2015), being in a similar broad range. The levels of most elements in the cultivated SW are within the ranges reported in the literature (Afonso et al., 2018). Only the concentration of Zn in the SW is below reported values in other cultivated green seaweeds of the genus *Ulva* (Afonso et al., 2018). Regarding the microalgal biomass, which corresponded to 100% of the feed in SW0, it is known that a balanced mineral content in the cultivation medium is required and largely reflected in the biomass composition (Gouveia, Marques, Sousa, Moura, & Bandarra, 2010).

The differences in the elemental composition of the feeds raise the issue of the extent to which the oysters were able to incorporate these elements into their tissues, which is answered in the next section.

### 3.2 Oyster elemental composition

The elemental composition of the studied oyster groups (Initial, SW100, SW50, SW25, and SW0) on a dry weight basis is presented in Table 3.

**Table 3:** Elemental composition (mg/kg dry weight and µg/kg dry weight) of the oysters at the beginning of the trial and after being fed different diets

Element	Initial	SW100	SW50	SW25	SW0
Li (mg/kg dw)	0.43 ± 0.01^a^	0.55 ± 0.01^b^	0.53 ± 0.04^b^	0.51 ± 0.02^b^	0.38 ± 0.01^a^
Be (µg/kg dw)	5 ± 0^b^	6 ± 0^a^	3 ± 0^c^	2 ± 0^c^	1 ± 0^d^
Cr (mg/kg dw)	1.00 ± 0.02^a^	1.73 ± 0.12^a^	0.92 ± 0.03^a^	0.91 ± 0.03^a^	n.d.
Mn (mg/kg dw)	54.4 ± 5.1^ab^	54.4 ± 0.6^a^	71.1 ± 7.0^b^	57.7 ± 2.6^ab^	57.2 ± 0.6^ab^
Co (µg/kg dw)	193 ± 2^a^	237 ± 3^ab^	272 ± 16^b^	261 ± 1^ab^	215 ± 5^ab^
Ni (mg/kg dw)	0.36 ± 0.01^ab^	0.57 ± 0.01^a^	0.29 ± 0.01^ab^	0.40 ± 0.02^ab^	0.18 ± 0.00^b^
Cu (mg/kg dw)	110.1 ± 3.7^a^	202.9 ± 0.4^c^	127.9 ± 4.1^b^	133.3 ± 4.0^b^	101.5 ± 0.3^a^
Zn (mg/kg dw)	1,220 ± 20^c^	2,716 ± 56^a^	1,331 ± 51^b^	1,330 ± 24^b^	743 ± 3^d^
As (mg/kg dw)	22.18 ± 2.03^a^	20.80 ± 0.55^a^	14.10 ± 0.23^b^	13.30 ± 0.88^b^	9.28 ± 0.02^c^
Se (mg/kg dw)	2.65 ± 0.14^a^	1.95 ± 0.02^b^	2.12 ± 0.06^b^	1.50 ± 0.08^c^	1.14 ± 0.07^d^
Br (mg/kg dw)	201 ± 5^a^	208 ± 4^a^	212 ± 2^a^	220 ± 5^a^	206 ± 14^a^
Sr (mg/kg dw)	21.5 ± 0.7^c^	35.8 ± 0.9^a^	26.1 ± 0.6^b^	26.8 ± 0.7^b^	18.3 ± 0.3^d^
Mo (mg/kg dw)	0.35 ± 0.01^ab^	0.63 ± 0.06^a^	0.23 ± 0.02^b^	0.35 ± 0.00^ab^	0.45 ± 0.01^ab^
Cd (mg/kg dw)	1.35 ± 0.01^cd^	2.84 ± 0.12^a^	1.50 ± 0.10^bc^	1.68 ± 0.09^b^	1.21 ± 0.02^d^
Sn (µg/kg dw)	33 ± 1^a^	32 ± 1^a^	27 ± 2^b^	28 ± 2^b^	16 ± 1^c^
I (mg/kg dw)	3.1 ± 0.0^a^	2.9 ± 0.0^ab^	2.2 ± 0.0^ab^	2.0 ± 0.1^ab^	1.6 ± 0.1^b^
Tl (µg/kg dw)	3 ± 0^a^	2 ± 0^ab^	2 ± 0^ab^	2 ± 0^ab^	1 ± 0^b^
Pb (mg/kg dw)	4.15 ± 0.25^a^	1.17 ± 0.03^a^	0.45 ± 0.01^a^	0.43 ± 0.02^a^	0.18 ± 0.00^a^

Abbreviation: n.d., not determined.

Values are presented as average ± *SD*. Different letters within a row correspond to statistical differences (*p* < 0.05).

First, a comparison with the elemental composition of the feeds shows that with exception of Cu and Zn, no accumulation of the studied elements was observed in the oysters’ meat. Quite the opposite was verified; most elemental contents were lower in the oysters than in the feeds. While, in the case of Cu, a factor of 10 expressed a large accumulation of this element by the oysters in all diets, in the case of Zn, factors in the range of 20–300 meant an even more intense accumulation. In the latter case, accumulation factor depended clearly on the feed given to the oyster, since this factor reached its highest value in the case of SW100 and its lowest value with the 100% microalgal feed (SW0).

Important effects may also be highlighted by a comparison between the initial group of oysters (Initial) and the various oyster groups after conditioning (SW100, SW50, SW25, and SW0). For several elements, there were differences between these groups. More specifically, whereas no statistically significant difference was observed for Cr (this element was not determined in one group, SW0), Br, and Pb contents, all other elements displayed different contents. The oysters fed SW100 had the highest levels of Be, Cu, Zn, Sr, and Cd. In the case of Zn, a content of 2,716 ± 56 mg/kg dw was determined. There was one instance where SW50 had a highest content than SW100: for Mn, SW50 had 71.1 ± 7.0 mg/kg dw and SW100 contained 54.4 ± 0.6 mg/kg dw. This was quite exceptional, since the most meaningful trend in the oysters throughout the various studied elements was a concentration decline with progressively lower levels of SW substitution for microalgae in the feeds. Indeed, the lowest elemental contents of Li, Be, Ni, Cu, Zn, As, Se, Sr, Cd, and Sn among the final groups were all registered for the oysters fed SW0. Moreover, if all five groups were considered (including the Initial group), the SW0 group had the lowest levels of Be, Zn, As, Se, Sr, and Sn. The Initial group had the highest Se content, 2.65 ± 0.14 mg/kg dw, and occupied a middle position in the concentration ranking of many elements. In fact, whereas many elemental concentrations increased from the Initial group to the final group SW100 (with SW addition to the feed), namely the concentrations of Li, Be, Cu, Zn, Sr, and Cd, the concentrations of Be, Zn, As, Se, Sr, Sn, I, and Tl declined from the Initial group to the final group SW0 (with microalgal addition to the feed).

The accumulation potential of mineral elements by bivalves is well known (Amiard et al., 2008; García‐Rico & Ruiz, 2001; Pavlov, Bezuidenhout, Frontasyeva, & Goryainova, 2015), and, in particular, oyster meat is typically rich in both macro elements and trace metals (Asha, Anandan, Mathew, & Lakshmanan, 2014). For instance, Indian backwater oyster (*Crassostrea madrasensis*) contained substantial levels of Zn and Se (Chakraborty, Chakkalakal, Joseph, & Joy, 2016). The Se levels reported for *C. madrasensis* were similar to those determined in the current study on *C. gigas*, being in the 0.5–3.0 mg/kg dw range (Chakraborty et al., 2016). However, Zn levels were much lower in *C. madrasensis* than in the conditioned *C. gigas*, not surpassing 270 mg/kg dw (Chakraborty et al., 2016), which is up to 10 times less than Zn content in the SW100 group. A study on cultivated *C. gigas* (García‐Rico & Ruiz, 2001) also found lower levels of Zn as well as of Cu and As in comparison with the present study. On the other hand, Cd levels were higher and Se and Pb ranges overlapped those values in the present study (García‐Rico & Ruiz, 2001). High Zn levels as for SW100 have been reported for *C. palmula* from Mexico, approximately 3,000 mg/kg dw (Páez‐Osuna, Osuna‐López, Izaguirre‐Fierro, & Zazueta‐Padilla, 1993). These oysters also had Cr levels similar to those in the current study. Besides, Cu reached 500 mg/kg dw in *C. palmula*, thus surpassing SW100 (Páez‐Osuna et al., 1993). Very high levels of Cd and Ni in *C. palmula*, which exceeded those in current study, were also reported. However, even in this case, Mn levels were below 40 mg/kg dw (Páez‐Osuna et al., 1993) and, as such, lower than any in the studied oyster groups. It should be remarked that *C. palmula* were Mexican oysters from a coastal lagoon associated with an agricultural drainage basin. A further comparison to wild *C. gigas* (Burioli et al., 2017) showed that the measured levels of Mn, Cu, Zn, As, Sn, Tl, and Pb in the current study were within the range reported for samples from marine ecosystems in Italy. Regarding the remaining elements, Sr and Br contents were similar to the values reported for *C. gigas* in the South African Atlantic Coast, 45 ± 5 mg/kg dw and 127 ± 10 mg/kg dw, respectively. In this study, the I content was lower than that of the South African oysters, 10.9 ± 0.4 mg/kg dw (Pavlov et al., 2015). This is noteworthy, especially for SW100 oysters, which were fed a SW feed rich in I (44.6 ± 0.8 mg/kg dw). Therefore, it seems that I assimilation by *C. gigas* was not very efficient during broodstock conditioning.

This assessment of assimilation of different elements leads to a general conclusion that SW0 oysters exhibit the lowest rates of accumulation of all studied elements. These results show that SW0 feed composition (only microalgal biomass)—which was richer in some elements than the other feeds, as it was the case for Cr, Mn, Co, Zn, Se, Sr, Cd, and Tl—did not affect the oyster meat composition. This phenomenon does not seem to result from a lower metabolic rate in the SW0 oysters. A low metabolic rate is sometimes an explanation for low accumulation rates (Pavlov et al., 2015). Indeed, the viability results associated with this study on broodstock conditioning of *C. gigas* (Rato et al., 2018) have shown that SW100 oysters exhibited a higher mortality when compared to SW0 and the remaining feeds, being unable to meet the demands for maintenance—a lower standard metabolic rate was measured in the SW100 oysters than in SW0 oysters (Ozório et al., 2018). Hence, a more satisfactory explanatory hypothesis would be differences in the growth rates between the various SW groups. A slower growth rate in SW100 oysters would result in higher levels of accumulated elements than in SW0 oysters (Pavlov et al., 2015). For the cases, where no differences in accumulation rates between SW0 and SW100 were detected (Mn, Co, Br, Mo, I, Tl, and Pb), also no differences between the SW100 and the Initial groups were found, thus signaling that probably oysters were not very efficient at accumulating these elements, as already hypothesized for I (see above).

The concentrations of some studied elements represent a nutritional advantage of oyster consumption. Indeed, some elements, such as Mn, Fe, Cu, Zn, and Se, act as cofactors for enzymatic reactions that are crucial in metabolism (Flemming, 1989), and Se has been associated with protection against oxidative stress, strengthening of the immune system, and regulation of growth and development (Rayman, 2000). Moreover, Se is a natural antagonist for mercury, thereby counteracting or eliminating symptoms of high exposures to this contaminant (Ralston & Raymond, 2010). Concerning I, it may have an antitumoural effect (Garcia‐Solis et al., 2005) and is required by humans for normal thyroid function (Dunn, 2003).

However, excessive intake of any of the studied elements may pose health risks. For this reason, there are upper limits. According to European Union legislation (European Commission, 2006), the maximum limit for toxic metals in edible wet weight of bivalve mollusks is 1.0 mg/kg for Cd and 1.5 mg/kg for Pb. These limits were converted to dry weight, assuming 85% moisture content in oysters (Volety, 2008; Watling & Watling, 1976), yielding 6.7 mg/kg dw for Cd and 10 mg/kg for Pb. Comparison with the present study's results (Table 3) shows that either a SW‐rich feed or a microalgal‐rich feed did not lead to Cd or Pb hazards in the oyster meat. This issue may also be relevant for essential metals, such as Cu and, even more, Zn, which accumulated to a large extent in oyster meat. It has been claimed that though Zn toxicity is rare, it can be toxic above the limit of 50 mg/kg ww (Hussein & Khaled, 2014), which converts to 335 mg/kg dw (Volety, 2008; Watling & Watling, 1976). Indeed, all oysters (Initial, SW100, SW50, SW25, and SW0) had Zn levels above this value, thus warranting further study and monitoring.

Finally, when discussing the elemental composition of the oyster meat, it must be taken into account that it is quite possible that only a minor share of the initial element contents becomes available for absorption across the intestinal wall after digestion, that is, bioaccessible (Afonso et al., 2015). Hence, in such a study as the current one, it is very important to assess the elemental bioaccessibility.

### 3.3 Elemental bioaccessibility

The bioaccessible elemental contents are presented in Table 4, and the bioaccessibility factors (in percentage) associated with each element are shown in Table 5.

**Table 4:** Bioaccessible elemental contents (mg/kg; calculated taking into account the mass of sample input in the in vitro digestion and subtracting blank interference) of the oysters at the beginning of the trial and after being fed different diets

Element	Initial	SW100	SW50	SW25	SW0
Mn (mg/kg)	8.7 ± 0.8^a^	8.6 ± 0.1^a^	19.6 ± 1.9^c^	13.0 ± 0.6^b^	16.7 ± 0.2^bc^
Cu (mg/kg)	106.1 ± 3.5^a^	222.4 ± 0.4^c^	141.5 ± 4.5^b^	149.0 ± 4.4^b^	107.4 ± 0.3^a^
Br (mg/kg)	200 ± 5^a^	205 ± 4^a^	208 ± 2^a^	218 ± 5^a^	204 ± 14^a^
Sr (mg/kg)	20.8 ± 0.7^ab^	26.2 ± 0.7^d^	23.1 ± 0.6^bc^	24.5 ± 0.7^cd^	20.3 ± 0.3^a^
Cd (mg/kg)	1.10 ± 0.00^a^	2.08 ± 0.08^d^	1.50 ± 0.10^bc^	1.63 ± 0.09^c^	1.44 ± 0.02^b^
I (mg/kg)	3.0 ± 0.0^a^	2.8 ± 0.0^a^	2.0 ± 0.0^b^	2.0 ± 0.1^b^	1.5 ± 0.1^c^
Pb (mg/kg)	2.06 ± 0.12^a^	0.66 ± 0.02^b^	0.35 ± 0.01^c^	0.27 ± 0.01^c^	0.20 ± 0.00^c^

Note

Values are presented as average ± *SD*. Different letters within a row correspond to statistical differences (*p* < 0.05).

**Table 5:** Elemental bioaccessibility (%) of the oysters at the beginning of the trial and after being fed different diets

Element	Initial	SW100	SW50	SW25	SW0
Mn	16 ± 0^a^	16 ± 0^a^	28 ± 2^bc^	23 ± 1^b^	29 ± 2^c^
Cu	96 ± 5^a^	110 ± 8^a^	111 ± 5^a^	112 ± 7^a^	106 ± 7^a^
Br	99 ± 0^a^	99 ± 1^a^	98 ± 1^a^	99 ± 1^a^	99 ± 1^a^
Sr	97 ± 2^bc^	73 ± 10^a^	89 ± 2^ab^	91 ± 0^abc^	111 ± 5^c^
Cd	81 ± 0^a^	73 ± 2^a^	100 ± 2^b^	97 ± 3^b^	108 ± 6^c^
I	98 ± 0^a^	97 ± 3^a^	93 ± 2^a^	98 ± 3^a^	94 ± 1^a^
Pb	50 ± 7^a^	56 ± 5^a^	79 ± 22^ab^	63 ± 5^a^	111 ± 9^b^

Note

Values are presented as average ± *SD*. Different letters within a row correspond to statistical differences (*p* < 0.05).

For some elements (Li, Be, Cr, Co, Ni, Se, Mo, Sn, and Tl), the very low bioaccessible contents near the limits of quantification and the interference of the blank (enzyme solutions used in the digestion model) did not enable a reliable determination of bioaccessibility percentages. In the case of Zn and As, there were also interference problems that affected the accuracy of the bioaccessible contents. Hence, only bioaccessible contents (and bioaccessibility percentages) for Mn, Cu, Br, Sr, Cd, I, and Pb are presented.

There was an increase of the bioaccessible concentration of Mn from the Initial group to the final group SW50 (with 50% SW and 50% microalgal addition to the feed), which was stronger than in the comparison of the total (before digestion) concentrations, and, to a lesser extent, to the final groups SW25 and SW0. For Cu, Br, Sr, and Cd, the bioaccessible contents varied between groups in a broadly similar fashion to that observed for the total (before digestion) contents. In the cases of Cu, Sr, and Cd, the largest bioaccessible content increases occurred from the Initial to the final group SW100. Br bioaccessible contents did not vary across all groups. Bioaccessible I levels declined from the Initial group to the final groups SW50 and SW25 and, even more intensely, to the final group SW0. For Pb, bioaccessible content declined from the Initial group (2.06 ± 0.12 mg/kg) to all final groups, whose Pb content was below 1 mg/kg.

These concentrations enabled the calculation of the bioaccessibility factors for each element in the studied oyster groups. The most striking feature of these bioaccessibility percentages was their similarity, hovering around 100% in the cases of Cu, Br, and I. For Sr and Cd, there were some differences, but values were also mostly near 100%. Only for Mn and Pb, bioaccessibility percentages deviated more from 100%. Indeed, the value for Pb was as low as 50% ± 7% (Initial group), and for Mn, all values were equal or lower than 29% ± 2% (SW0). For the cases where differences were detected between oyster groups, it was observed that bioaccessibility increased from the Initial and SW100 groups to the SW0 group, namely in Mn, Cd, and Pb. For Sr, bioaccessibility of the SW0 group was higher than that of the SW100 group, but not higher than that of the Initial group.

There are only a few studies on elemental bioaccessibility in oysters (Amiard et al., 2008; He & Wang, 2013). In spite of some methodological differences, the in vitro digestion models are comparable, at least the static models used in these two experimental works. These studies show a large variability of bioaccessibility percentages across elements. Indeed, in *C. gigas* from the French Atlantic Coast, while Pb bioaccessibility was lower than 50%, Cu and Cd bioaccessibility was higher than 80% (Amiard et al., 2008). For the latter elements, there is considerable agreement with current study's results (Table 5). In the case of Pb, whereas there was similarity with Initial and SW100 groups’ results, the SW0 results were clearly higher. Furthermore, *C. gigas* from Chinese coastal waters had high Mn, Cu, and Cd bioaccessibility, 80%–95%, but a somewhat lower Pb bioaccessibility of 69.4% ± 2.3% (He & Wang, 2013). Again, for Cu and Cd, there was agreement with the current study, whereas, for Mn, values were higher than in Initial/SW100/ SW50/SW25/SW0 oysters.

Different explanations can be put forward for the results. In any case, a possible correlation between bioaccessibility and total concentration of all studied elements is not supported by the data—a comparison between Mn, Cu, and Cd refutes any such correlation. Though there is some correlation between decreasing Sr content (e.g., SW100 vs. SW0) (Table 3) and increasing bioaccessibility percentages (Table 5), there are not enough data to statistically support such possibility. In fact, other authors have also failed to find such correlations (He & Wang, 2013). A relationship between bioaccessibility and elemental distribution among organs or even within cells may also be a working hypothesis for explaining differences between elements. The relatively low Pb bioaccessibility may be related to its mode of storage. It has been pointed out that Pb is present in numerous cell types in molluskan tissues, being distributed both in lysosomes and cytosol (Marigomez, Soto, Carajaville, Angulo, & Giambérini, 2002). More specifically, in *C. gigas*, lysosomes are the major intracellular structures storing Pb in the gills, digestive tract, and digestive gland (Amiard et al., 2008). The Pb bioaccessibility increase in SW0 oysters could be related to a different distribution, but the intermediate groups SW50/SW25 show no clear trend. As a third hypothesis, the trend to higher elemental bioaccessibility in SW0 versus SW100 (Mn, Sr, Cd, and Pb) may be related to a feed effect on the chemical form or association to specific molecules of each element. This means that further study is warranted, including bioavailability assessment through in vivo assays.

## 4 CONCLUSIONS

This study provided important information on the elemental composition and bioaccessibility of oysters fed diets with variable levels of seaweed substitution for microalgal biomass, which is highly relevant in culture conditioning. Namely, it was observed that oysters fed only seaweed had the highest levels of Be, Cu, Zn, Sr, and Cd. The most important trend in the oysters’ elemental composition was a concentration decline with progressively lower levels of seaweed substitution for microalgae in the feeds. Whereas Li, Be, Cu, Zn, Sr, and Cd concentrations increased from the Initial group to the final group of oysters fed only seaweed, the concentrations of Be, Zn, As, Se, Sr, Sn, I, and Tl declined from the Initial group to the final group of oysters fed only microalgae. No Cd or Pb hazard was found in oyster meat. However, for Zn, given the high observed levels in the studied oysters, some caution and further study are warranted. Although there was some variation in elemental bioaccessibility, its Cu, Br, Sr, Cd, and I values were near 100%. On the other hand, the bioaccessibility of Pb was 50% ± 7% (Initial group) and that of Mn was equal or lower than 29% ± 2% (microalgal feed). It was observed that Mn, Cd, and Pb bioaccessibility increased from the Initial and seaweed feed groups to the microalgal feed group.

## CONFLICT OF INTEREST

The authors declare that they do not have any conflict of interest.

## ETHICAL APPROVAL

The animal (Pacific oyster) testing fulfilled all requirements resulting from the regulations concerning animal experimentation.
